# Determination of the lactose and galactose content of common foods: Relevance to galactosemia

**DOI:** 10.1002/fsn3.2976

**Published:** 2022-07-19

**Authors:** Loai A. Shakerdi, Leonie Wallace, Georgina Smyth, Nora Madden, Anne Clark, Una Hendroff, Marianne McGovern, Sarah Connellan, Barbara Gillman, Eileen P. Treacy

**Affiliations:** ^1^ National Centre for Inherited Metabolic Disorders Mater Misericordiae University Hospital Dublin 7 Ireland; ^2^ Public Analyst's Laboratory Galway Ireland; ^3^ National Centre for Inherited Metabolic Disorders Children's Health Ireland (CHI) at Temple Street Dublin 1 Ireland; ^4^ School of Medicine Trinity College Dublin Dublin 2 Ireland; ^5^ UCD School of Medicine University College Dublin Dublin 4 Ireland

**Keywords:** cheese, galactose, Galactosemia, GALT, lactose

## Abstract

Classical galactosemia (CG) is a disorder of galactose metabolism which results from deficiency of the enzyme galactose‐1‐phosphate uridylyl transferase (GALT). Treatment consists of immediately eliminating galactose from the diet in the new‐born and lifelong restriction of dietary galactose. The inclusion of a wider variety of foods for people with CG may provide many benefits, including improved nutritional adequacy and quality of life. Galactose plays an important role in glycosylation of glycoproteins and glycolipids. Moderate liberalization of galactose restriction has been shown to improve immunoglobulin G (IgG) glycosylation for some individuals with CG. Moreover, recent outcome research suggests that strict restriction of nondairy galactose may have more unfavorable outcomes than moderate liberalization in CG patients. In the current work, based on patient feedback, we have analyzed the lactose and galactose content of different foods available in Ireland. These include a range of cheeses, yogurts, pizzas, soups, biscuits, cakes, pastries, crackers, mayonnaises, salad creams, fat spreads, crisps, corn chips, salamis, and gravies. This work provides information to support the development of a practical food‐based approach to facilitate analysis of dietary galactose intake and to possibly increase overall variety of food choices for people with CG.

## Graphical abstract

In this work we have analysed the galactose content of commonly available foods in Ireland to provide more accurate information on galactose and lactose contents. This information is heretofore lacking and will provide valuable information to the international Galactosaemia community; and also, for researchers and clinicians in the field of Human Nutrition.

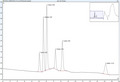

## 1 INTRODUCTION

Classical galactosemia (CG) (OMIM # 230400) is a disorder of galactose metabolism which results from deficiency of the enzyme galactose‐1‐phosphate uridylyl transferase (GALT), a central enzyme in the highly conserved Leloir pathway of galactose metabolism (Isselbacher et al., 1956). This enzymatic defect leads to a build‐up of galactose‐1‐phosphate (Gal‐1‐P) and the accumulation of galactose and other by‐products causing life‐threatening symptoms including lethargy, feeding problems, hypoglycemia, hepatomegaly, jaundice, ascites, diarrhea, bleeding diathesis, and *Escherichia coli* (*E. coli*) sepsis (Rubio‐Gozalbo et al., 2019).

The CG has a prevalence in Western countries of between 1:16,000 and 1:60,000 live births (Rubio‐Gozalbo et al., 2019). Since 1982, the average live birth incidence rate of CG in the Irish population is approximately 1:16,476 births. This reflects a high incidence in the Irish Traveler population, with an estimated birth incidence of 1:33,917 in the non‐Traveler Irish population (Coss et al., 2013).

Diagnosis is established by measuring galactose or its metabolites such as galactose‐1‐phosphate (Gal‐1‐P) and galactitol, in blood or urine. The gold standard for the diagnosis of CG is the measurement of GALT activity in red blood cells (RBCs) (Coelho et al., 2017).

Treatment consists of immediately eliminating galactose from the diet in the new‐born and for lifelong strict restriction of dietary galactose. Although this intervention is life‐saving in the new‐born, there is abundant evidence internationally of long‐term complications in treated patients that include cognitive impairment, speech abnormalities, neurological abnormalities, behavior abnormalities, and a very high prevalence of primary ovarian insufficiency in affected females (Colhoun et al., 2018; Fridovich‐Keil et al., 2011; Rubio‐Gozalbo et al., 2010). The UK Steering Group on Galactosaemia in 1999 proposed that a strictly restricted galactose diet should be followed for individuals affected with CG (Walter et al., 1999). Historically, the diet for children with CG was not as strict at the age they entered the junior school system at approximately 5–6 years old (Francis, 1974). The diet remained free from dairy products, although food products containing traces of lactose/galactose were consumed in order to manage this diet within a real‐world environment.

International clinical guidelines for the management of CG were published in 2017 (Welling et al., 2017). The Guideline Taskforce recommended treating CG patients with a lifelong galactose‐restricted diet that only eliminates sources of lactose and galactose from dairy products. There was insufficient evidence to support a specific age‐related recommendation for the quantity of galactose allowed in the diet.

This guideline permits galactose from non‐milk sources that contribute minimal dietary galactose with the inclusion of small amounts of galactose present in specific mature cheeses and caseinates. It also recommended that any amount and type of fruits, vegetables, legumes, unfermented soy‐based products, and the food additives sodium or calcium caseinate could be included in the diet of CG patients (Welling et al., 2017).

Galactose plays an important role in glycosylation of glycoproteins and glycolipids (Pucic et al., 2011). Moderate liberalization of galactose diet has been shown to improve immunoglobulin (IgG) glycosylation and to be associated with improved glycosylation in a small group of children and in adults (Coss et al., 2012; Treacy et al., 2021). Recently, a retrospective dietary analysis of 231 CG patients showed no significant association between non‐dairy galactose restriction in early childhood and the outcomes studied (including growth, adaptive behaviors, receipt of speech therapy, or educational assistance) (Frederick et al., 2017). Subjects with a higher galactose intake did not exhibit an increased incidence of complications and those who were very compliant with dietary restrictions did not have more favorable outcomes (Hughes et al., 2009). Outcome studies performed by our own group, and others, have indicated that the severity of dietary galactose restriction is not associated with better cognitive and neurological outcomes. The studies indicated the converse, i.e., that over‐restriction of dietary galactose might be harmful (Hughes et al., 2009; Jumbo‐Lucioni et al., 2012), with further evidence suggesting that strict restriction of non‐dairy galactose may possibly have sub‐optimal rather than beneficial outcomes (Rubio‐Gozalbo et al., 2019; Schweitzer et al., 1993; Waisbren et al., 2012). In the recently reported GalNet (The Galactosemia Network) registry outcome study of over 500 CG patients, it was noted that patients following a strict galactose‐restricted diet (lactose, fruit, and vegetables restricted) developed neurological complications more frequently than patients with a less strict diet (Rubio‐Gozalbo et al., 2019).

Galactose is derived from the breakdown of lactose. Lactose is the disaccharide of milk and milk‐containing products. Lactose is hydrolyzed to glucose and galactose by lactase in humans (Reichardt & Berg, 1988; Valle et al., 2019). During the process of digestion, lactose is broken down in the ratio of 47.37% glucose:52.63% galactose. Therefore, 1 g or 1000 mg lactose = 526 mg ~ 500 mg galactose by extrapolation (Ohlsson et al., 2017). On a galactose‐restricted diet, endogenous galactose production ranges from 1.1 to 1.3 g/day (Liu et al., 2000). It has been estimated that the rate of de novo synthesis of galactose, in healthy and in subjects with galactosemia, ranges from 0.48 to 1.71 mg/kg/h (Berry et al., 2004). For an average adult, weighing 70 kg, this is equivalent to 800–2873 mg galactose per day. Endogenous production of galactose was not found to be affected by the exogenous intake of lactose and galactose from the diet (Huidekoper et al., 2005).

It has also been shown that the daily ingestion of 200 mg of fruit‐derived galactose had no impact on RBC Gal‐1‐P values and relatively little effect on urinary galactitol levels in patients with null RBC GALT activity (Coelho et al., 2017; Berry et al., 1993). This may suggest that endogenous galactose production is the main source of galactose metabolites routinely detected in patients on lactose‐restricted diets (Berry et al., 1993). Subsequently, it has been noted that there is significant endogenous galactose production in children and adults (Berry et al., 2004; Schadewaldt et al., 2004). Thus, foods such as offal, fruits, legumes, and pulses are insignificant sources of galactose compared with endogenous production and there is no evidence to support their restriction (Berry et al., 1995). Furthermore, a number of commonly consumed foods have been found to have reduced galactose content following analysis and have therefore been suggested for inclusion in a galactose‐restricted diet. These include lactose‐hydrolyzed milk fermented using a traditional kefir culture (Varga et al., 2006), Pecorino Romano sheep cheese (Idda et al., 2018), Gruyere, Emmental, Jarlsberg, Italian Parmesan (Parmigiano Reggiano and Grana Padano), Comte, Emmi Swiss Fondue, and specific British brands of mature cheddar cheese. However, the galactose content of processed foods can vary significantly as thermal changes, clarification aid, and enzymatic liquefaction aid may contribute to altered galactose content during food processing (Scaman et al., 2004).

Significant improvement in proteins and lipids glycosylation was demonstrated with moderate galactose diet liberalization in a subset of adults and children, specifically allowing up to 500 mg of galactose in the pediatric study and with beneficial glycosylation effects up to 1000 to 2000 mg of galactose in adults (Lee et al., 2003; Maratha et al., 2017; Panis et al., 2006). Bosch et al., in a study of adolescents with galactosemia, noted that a daily dietary intake of 500 mg of galactose was well tolerated (Bosch, 2011). There are also reports on individual adult CG patients who self‐liberalized their diets without adverse consequences. No physical, ophthalmological, or biochemical abnormalities were detected in three adolescents who ingested up to 600 mg of galactose per day for 6 weeks (Bosch et al., 2004). When these cases were compared to adherent patients, they reported a better quality of life (Lee et al., 2003; Panis et al., 2006).

## 2 METHODS

### 2.1 Patient surveys

An anonymous patient questionnaire was sent to all CG patients or their carers attending the National Centre for Inherited Metabolic Disorders, Temple Street Hospital, Dublin in 2012. This included a request to list foods that patients would like analyzed to ascertain if these products contained sufficiently minimal amounts of galactose to be allowed in the diet. Information from this questionnaire was used to perform a phone survey involving 13 adults and 8 children with CG in 2015 (Appendix S1). Informed verbal consent was obtained from all patients prior to administering the questionnaire.

### 2.2 Chemical analysis of lactose and galactose in the food samples using high‐performance anion‐exchange chromatography/pulsed amperometric detection

Food selected for analysis from the phone survey were all analyzed between 2016 and 2021, at the Public Analyst's Laboratory, Galway, using high‐performance anion‐exchange chromatography/pulsed amperometric detection (HPAEC/PAD) to accurately determine galactose and lactose concentrations in food products. This analytical method is an in‐house developed method and is accredited to ISO 17025 since November 2019. The analysis accurately determines sucrose, galactose, glucose, fructose, lactose, and maltose concentrations in beverages and foods. Food brand names were anonymized and individual samples were labeled by a laboratory reference number in all results tables (see Appendix S1). Food samples were mixed, homogenized, or grated (in the case of hard cheese finely grated) before analysis. A test portion was extracted with carrez solution. Some food matrices, i.e., pizza and cheese etc. required an Ultra‐Turrax step with water prior to the carrez extraction step and emulsified samples (i.e., butters, spreads, mayonnaises, etc.) were de‐emulsified prior to the carrez extraction step, by placing a test portion with water in a water bath set at 45°C for 5–30 min. The sample extract was vortexed for ~15 s to thoroughly mix the contents and centrifuged at 3000 rpm for 20 min at ambient temperature and filtered. The filtered extract was then analyzed using HPAEC/PAD to accurately determine galactose and lactose concentrations in foods. The method is performed using a Thermo Scientific Dionex ICS 5000+ HPIC system using a Dionex CarboPac SA10‐4 μm (4 × 250 mm) Column with a Dionex CarboPac SA10G‐4 μm (4 × 50 mm) guard column and a Dionex IonPac NG1 column (4 × 35 mm).

## 3 RESULTS

### 3.1 Patient survey

The results of the phone survey were reviewed by two clinical dietitians and the following food groups were chosen for analysis;

- Cheeses
- Yogurts
- Pizzas
- Soups
- Biscuits
- Crackers
- Cakes and pastries
- Mayonnaise and salad creams
- Fat spreads
- Crisps and corn chips
- Salamis
- Gravies

### 3.2 HPAEC/PAD method analytics

**FIGURE 1:**
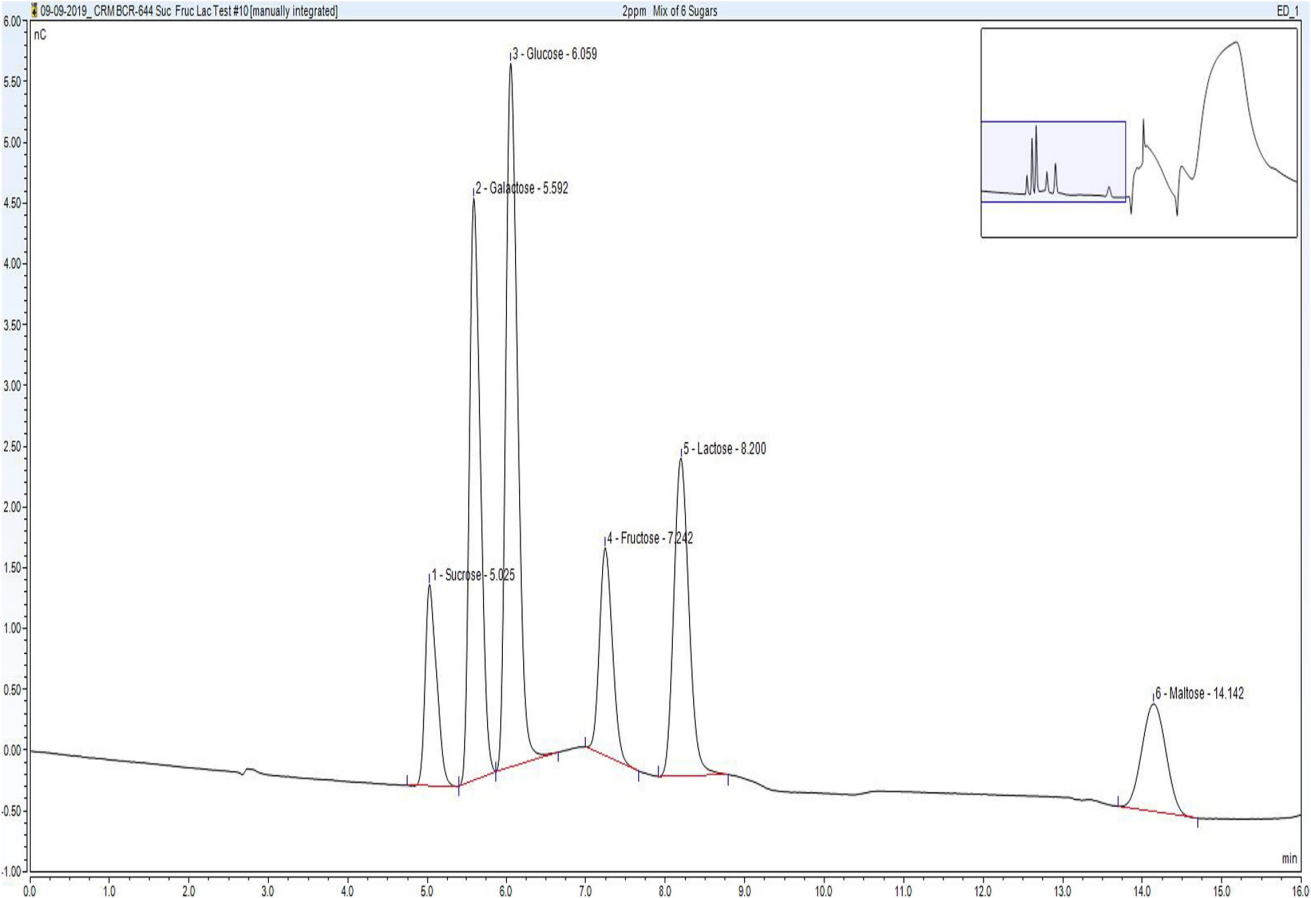
Chromatogram of ~2 mg/L standard. The y axis represents the response/signal and the x axis represents the retention time (min)

Spiking recovery studies were carried out for all food types. Recovery data for galactose are: range: (88–111%), mean: 98%, standard deviation (SD): 7%. Recovery data for lactose are: range: (85–112%), mean: 97%, SD: 6%. For more information, see Appendix S1 (Table S1). The laboratory has participated in the FAPAS Proficiency Test (PT) scheme for sugars analysis from September 2015 to July 2020. Satisfactory z‐scores (−2.0 to 2.0) have been obtained for all rounds. The PT data for galactose and lactose can be found in the Appendix S1 (Table S2). The average recovery for galactose is 100% (assigned value range: 0.93–4.23 g/100 g). The average recovery for lactose is 105% (assigned value range: 1.67–16.4 g/100 g) (Table S3 – Appendix S1). A chromatogram of ~2 mg/L standard is shown in (Figure 1 – Appendix S1).

### 3.3 Food analysis

Results are presented in ascending total galactose content (mg/100 g) for all food groups. Minimum and maximum values for galactose, lactose, “released” galactose, total galactose, portion size, and total galactose per portion are included for each food type.

Table **1** provides an overview of all foods analyzed. The analysis of dairy and dairy alternatives included 35 cheeses (Table S4a). One sample was used for each cheese, with the exception of one type of lactose‐free cheese (two samples [F145‐2016, F157‐2016] from one batch analyzed); one type of dairy‐free cheese (two samples [F150, F716] from two different batches) and one type of mature white cheddar cheese from a supermarket brand (six samples: F158‐2016, F714‐2016, F755‐2016, F756‐2016, F757‐2016, and F758‐2016 from six different batches). Of the 35 cheeses, 16 had a galactose content <25 mg/100 g. These included four dairy‐free cheese alternatives, six types of mature cheddars (four white and two red), two Dutch semi‐hard cheeses including a popular miniature snack cheese, and four non‐cheddar white cheeses including two reduced fat white cheeses and one vintage. Of the six mature cheddars with galactose <25 mg/100 g, two were of the same type and brand but from different batches. The remaining six samples from this cheese had galactose contents ranging from 42 to 255 mg galactose per 100 g. The remaining seven mature cheddar cheeses had galactose contents ranging from 32 to 237 mg/100 g. Two non‐mature cheddar cheeses from one brand were analyzed and were shown to have galactose contents of 193–283 mg/100 g. The cheeses with the highest galactose content were cheeses labeled “lactose‐free” (824–908 mg/100 g) and processed cheeses including a soft cheese block and cheese slices (721–‐2656 mg/100 g). This analysis confirms the suitability of dairy‐free cheeses and the unsuitability of both lactose‐free and highly processed cheeses/cheese spreads in a galactose‐restricted diet. Some mature cheddar cheeses were found to have minimal galactose content (<25 mg/100 g). However, the majority had a higher galactose content. A number of cheeses for which no previous analyses exist were included in this study, including two types of Dutch semi‐hard cow's milk cheese, reduced fat white cheeses, and one vintage cheese from a popular Irish brand. All of these cheeses had low galactose contents (<25 mg/100 g).

**TABLE 1:** Galactose and lactose contents of patient‐selected foods

Sample description	Sample size	Galactose (mg/100 g)	Lactose (mg/100 g)	“Released” galactose (from lactose) (mg/100 g)	Total galactose value (mg/100 g)	Portion (g)	Total galactose value (mg per portion)
a. Dairy and alternatives – Cheese							
Dairy free	4	<10	<10	<5	<15	30	<5
White (noncheddar)	4	<10–20	<10	<5	11–20	30	3–6
Dutch semihard	2	<10	<10	<5	<15	20–30	<5
Mature cheddar	17	<10–255	<10	<5	<15–255	30	<5–77
Nonmature cheddar	2	193–283	<10	<5	193–283	30	58–85
Processed	4	30–67	1247–4993	656–2626	721–2656	30	216–797
Lactose free	2	824–908	<10	<5	824–908	30	247–272
b. Dairy and alternatives – Yogurt							
Dairy free	7	<10	<10–94	<5–49	8–49	125	10–62
Fromage frais	3	727–970	1879–4833	988–2542	1934–3269	60–85	1644–1961
Goat's milk	2	1795–1900	103–131	54–69	1849–1969	90	1664–1772
Natural	2	705–1244	3832–4541	2016–2389	2721–3633	125	3401–4541
Flavored	3	757–872	1992–3113	1048–1637	1920–2440	125–160	3050–3072
Zero % Greek style (flavored & natural)	3	511–609	2176–2477	1145–1303	1656–1910	150	2483–2865
Low fat (flavored & natural)	3	835–1027	2699–4922	1420–2589	2269–3616	125–175	3903–4520
c. Cooked pizzas							
Ham & pineapple/Hawaiian	3	55–98	7–124	4–65	59–163	175–183	104–299
Pepperoni (± salami/±chorizo)	7	47–176	2–125	1–66	51–177	153–245	81–271
Cheese pizza (including four cheese)	6	70–214	2–159	1–84	71–216	153–209	110–358
d. Takeaway pizzas							
Ham & pineapple	4	90–201	4–328	2–173	132–308	250	330–769
Pepperoni	4	115–237	4–323	2–170	151–294	250	378–736
Cheese	4	125–268	4–374	2–197	168–350	250	420–874
e. Soups							
Dairy free (no milk ingredients)	3	3	<2	<1	3	200	6
Containing butter and cream as only milk ingredients	4	2‐ < 60	70–230	37–121	37–121	200	74–242
Containing other milk ingredients (e.g., skimmed milk powder)	8	2–82	160–1550	84–815	84–855	200	168–1711
f. Biscuits							
Dairy‐free^a^	7	<11‐45	<11–126	<6–66	6–111	8.3–16.7	<0.5–16.7
Containing butteroil as only milk ingredient	1	<11	159	84	84	12.2	10.25
Nonchocolate biscuits containing milk ingredients	4	<2–14	30–1103	16–580	16–594	12.5–28	2.5–104
Chocolate biscuits containing milk ingredients	2	<11–108	1929–5839	1015–3071	1015–3179	16.7–20.7	169.5–658.1
g. Crackers							
Dairy free^a^	5	<2–26	<2‐22	<1–12	<3–34	3.4–10.4	0.2–1.2
Crackers containing milk ingredients (cheese powder 5.4%)	1	9	210	110	119	3.6	4.3
h. Cakes and pastries							
Plain croissants (including loose samples with no ingredients listed and packaged sample with milk ingredients)	3	9–12	193–435	102–229	111–241	40–61	66–147
Other pastries (including loose samples with no ingredients listed and packaged sample with milk ingredients)	2	<2–4	20–113	11–59	11–63	59–69	6–44
Swiss rolls (containing skimmed milk powder as only milk ingredient)	2	4–10	415–868	218–457	222–467	36–37	82–168
Plain cakes (containing multiple milk ingredients including whey powder, skimmed milk powder, buttermilk powder)	2	13–16	413–583	217–307	230–323	27–39	62–126
Flavored cakes (containing multiple milk ingredients including whey powder, skimmed milk powder, milk protein)	5	<2–51	422–1600	222–842	222–842	26–103	71–335
i. Mayonnaise and salad cream							
Light & “real” mayonnaise with no milk ingredients listed	4	<25	<25	<13	<38	30	<11
Light mayonnaise with milk ingredients listed (cream powder)	2	<100	155–159	82–84	82–84	30	24–25
Salad cream (no milk ingredients listed)	2	<25	<25	<13	<38	30	<11
j. Fat spreads							
No milk ingredients listed	1	<10	<10	<5	<15	10	<2
Buttermilk as only milk ingredient	6	<10	23–438	12–230	12–230	10	1–23
Other milk ingredients (e.g., cream, whey powder)	2	<10	320–634	168–333	168–333	10	17–33
k. Crisps and chips							
Crisps & tortilla chips with no milk ingredients listed	5	<5	<5	<3	<8	25–34	<3
Potato crisps (milk ingredients listed)	7	<5–8	9–2610	5–1373	5–1373	25–34	1–343
Tortilla/corn chips (milk ingredients listed)	5	<5–21	346–1797	182–945	182–966	30–40	55–386
Baked potato snacks (milk ingredients listed)	2	<5‐<14	483–1422	254–748	254–748	25–30	76–187
Stackable potato‐based snacks (milk ingredients listed)	2	<5	24–89	13–47	13–47	30	4–14
l. Salamis							
Dairy free (Italian Milano/Smoked/German Style/Peppered)	4	<5	<5	<3	<8	15–30	<2
m. Gravies							
Dairy free (no milk ingredients)	14	<5	<5	<3	<8	50–75 (ml)	<0.4
Containing milk/milk proteins only	4	<5	<5	<3	<8	50 (ml)	<0.4
Containing milk/milk proteins and lactose	1	28	495	260	288	100 (ml)	22.2

^a^ No milk declared/may contain (traces of) milk/produced in a factory handling milk but on a different line/manufactured on equipment that handles milk).

Twenty‐three yogurt products were analyzed (Table S4b). All non‐dairy yogurts labeled “dairy‐free” (coconut and soya‐based yogurts) contained <50 mg galactose per 100 g and all dairy‐containing yogurts contained >1600 mg galactose/100 g. This analysis confirms the suitability of dairy‐free yogurts for patients avoiding galactose and provides more insight into the galactose contents of a wide range of dairy‐containing yogurts. As all dairy‐containing yogurts contained a significant amount of galactose, they remain unsuitable for most patients on a galactose‐restricted diet, unless included in very small, measured quantities as part of a galactose liberalized diet.

Table S4c,d outlines the analysis of 16 supermarket‐bought pizzas which were cooked prior to analysis. All pizzas contained dairy cheese and ranged in galactose content from 51 to 216 mg per 100 g. Pepperoni and ham & pineapple topped pizzas had a slightly lower galactose content than cheese‐only pizzas (51–177 mg compared to 71–216 mg per 100 g). The regular cheese pizza, pepperoni pizza, and ham & pineapple pizza from four popular franchise restaurants were also analyzed. All franchise pizzas contained dairy cheese and ranged in galactose content from 132 to 350 mg per 100 g. Pepperoni and ham & pineapple topped takeaway pizzas also had a slightly lower galactose content than cheese‐only takeaway pizzas (132–308 mg compared to 168–350 mg per 100 g) (Table S4d). Thus, all supermarket‐bought pizzas analyzed contained <400 mg galactose per portion (half a pizza). Takeaway pizzas tended to have higher galactose contents and are likely to be less standardized than supermarket‐bought pizzas. Our results also indicate that cheese pizzas are higher in galactose compared to pizzas with varied toppings (pepperoni, ham & pineapple).

Table S4e outlines the lactose and galactose contents of a range of soups (fresh and canned). All dairy‐free soups contained minimal galactose (3 mg galactose per 100 g) and soups containing butter and cream as the only milk ingredients had a galactose content of 37–121 mg per 100 g. Soups also containing other milk ingredients contained 84–855 mg galactose per 100 g, including “cream of” soups which ranged from 237 to 282 mg galactose per 100 g for cream of tomato (*n* = 3), 409–569 mg galactose per 100 g for cream of chicken (*n* = 2), and 855 mg galactose per 100 g for cream of mushroom (*n* = 1). Therefore, for patients advised to avoid galactose, dairy‐free soups are suitable, as all contained trace amounts of galactose. Soups containing butter and cream may be suitable for patients on a liberalized diet, as a serving size (400 g) would typically provide 148–484 mg of galactose. However, soups containing other milk ingredients and any soups labeled “cream of” contained higher amounts of galactose and would be unsuitable for patients with CG.

Fourteen types of biscuits were analyzed (Table S4f). Six out of seven dairy‐free biscuits (including those which may contain traces of milk) contained <25 g galactose per 100 g, while one biscuit (ginger‐flavored biscuits with a mallow center) had 111 mg galactose per 100 g. One biscuit (Jaffa cakes) had butteroil listed as the only milk ingredient and contained 84 mg galactose per 100 g. Non‐chocolate biscuits containing milk ingredients consisted of Viennese whirls, plain digestives, custard creams, and shortcake biscuits with a raspberry‐flavored apple jam which contained 16 mg, 17 mg, 93 mg, and 594 mg galactose per 100 g, respectively. Chocolate‐containing biscuits, which consisted of milk chocolate digestives and mocha flavor wafer fingers covered with milk chocolate, contained 1015 mg and 3179 mg galactose per 100 g, respectively. This analysis highlights a number of biscuits with minimal galactose content (<25 mg per 100 g or <4 mg per biscuit) including bourbon creams, digestives, pink wafers, oat‐based biscuits, rich teas, and ginger nuts. This includes one type of biscuit which contained dried skimmed milk (digestives). Conversely, one biscuit analyzed had no milk ingredients listed but had a relatively high galactose content (111 mg per 100 g). Chocolate‐containing biscuits had substantially higher galactose contents compared to plain versions and would therefore be unsuitable for inclusion in a galactose‐restricted diet.

Six types of crackers from five popular brands were analyzed (Table S4g). Five contained no dairy products and had low galactose contents (≤34 mg per 100 g). Per 100 g, oatcakes had the lowest galactose content (<3 mg), followed by salted savory snack biscuits (5 mg), cream crackers (10 mg), crackerbread (12 mg), and table water biscuits (34 mg). One cracker contained cheese powder and had a higher galactose content (119 mg per 100 g). However, per cracker, there was <5 mg galactose.

Fourteen types of cakes and pastries were analyzed (Table S4h). All cakes and pastries analyzed contained dairy ingredients such as whey powder and milk proteins, with the potential exception of three loose samples which did not have ingredients listed. Plain croissants, including all‐butter croissants with milk ingredients as well as two loose samples with no ingredients listed, contained 111–241 mg galactose per 100 g. Other pastries (Bramley apple pies and pain au chocolat) contained 11–63 mg per 100 g, while cakes contained 222–842 mg per 100 g. Thus, pastries tended to have a lower galactose content compared to cakes, with one product (Bramley apple pies) containing minimal galactose content (6 mg per portion), despite containing whey powder and milk proteins. While all cakes contained >200 mg galactose per 100 g, several cakes contained <100 mg galactose per portion (queen cakes, mini Battenburgs, and ripple Swiss rolls). Overall, all cakes and pastries contained <350 mg galactose per portion.

Three popular brands of light and “real” mayonnaise were analyzed (Table S4i). Two light mayonnaises contained milk cream powder and had a higher galactose content of 82–84 mg/100 g, compared to <38 mg galactose per 100 g for all non‐dairy containing mayonnaises. Two brands of salad cream were also analyzed (Table S4i). Neither brand contained any milk ingredients but both contained <38 mg galactose/100 g. Nine fat spreads including dairy‐free spreads, buttermilk‐containing spreads, and pure butter were analyzed (Table S4j). A dairy‐free baking block for cakes and pastry contained <15 mg galactose per 100 g and the galactose content of dairy‐containing spreads ranged from 12 to 333 mg per 100 g, with pure butter containing the most galactose (333 mg per 100 g or 33 mg per 10 g portion).

Twenty‐one types of potato crisps and tortilla/chips were analyzed (Table S4k). All crisps or chips containing no milk ingredients contained minimal galactose (<8 mg per 100 g). These included one brand of chili‐flavored corn chips, three brands of cheese and onion potato crisps, and one brand of salt and vinegar potato crisps. All other crisp/chip varieties contained milk ingredients such as cheese powder, dried skimmed milk, and yogurt powder. A wide range of dairy‐containing potato crisps were analyzed and total galactose contents per 100 g varied from 5 to 1373 mg. The potato crisp with the lowest galactose content was a supermarket brand of cheese and onion crisps containing vegetarian cheese powder and milk‐containing flavoring (5 mg galactose/100 g). The potato crisp with the highest galactose content was a supermarket brand of salt and vinegar crisps containing lactose and dried skimmed milk (1373 mg galactose/100 g). Three varieties of dairy‐containing corn/tortilla chips from three brands were analyzed. Per 100 g, “cool” flavor chips (*n* = 4) contained 182–966 mg galactose and cheese flavor chips (*n* = 1) contained 527 mg galactose per 100 g. Two flavors of baked crisps were analyzed from two brands. The sour cream and onion flavor (ingredients included soured cream powder and whey powder) contained 254 mg galactose per 100 g and the cheese and onion flavor (ingredients included whey permeate, whey protein, dried cheese, and skimmed milk powder) contained 748 mg galactose per 100 g. Two flavors from one popular brand of stackable potato‐based snacks were analyzed; the cheese and onion flavor contained 13 mg per 100 g and the sour cream and onion flavor contained 47 mg per 100 g. This analysis confirms the suitability of dairy‐free crisps (no milk ingredients) for a galactose‐restricted diet. Sixteen of the 21 crisps and tortilla/corn chips analyzed contained milk products, with the majority (Coss et al., 2012) of these containing <200 mg galactose per 25–34 g portion. The remaining four crisps/chips contained 241–386 mg galactose per portion.

Four types of salami, one each from four major supermarket brands, were analyzed (Table S4l). None of the salami products contained any milk products and all had minimal galactose contents (<8 mg per 100 g). Nineteen types of gravies from six brands were analyzed (Table S4m). Fourteen gravies had no milk ingredients and five contained milk, milk ingredients, lactose, and/or milk proteins. Eighteen gravies had minimal galactose content (<8 mg per 100 g) and one gravy had 288 mg galactose per 100 g. However, per 100 ml portion, there was only 22.2 mg galactose.

## 4 DISCUSSION

A galactose‐restricted diet has been the mainstay of therapy for management of galactosemia since 1935. The principle of dietary management of CG should be not only to restrict intoxication of galactose and its by‐products but also to allow enough substrate for normal growth and development while optimizing nutritional status. There is still an ongoing debate about the degree of restriction required in childhood and adulthood. Many patients with galactosemia, regardless of the degree of galactose restriction, are still at risk of developing long‐term complications, including cognitive delay, language impairment, reduced bone mass, and female infertility. Over‐restriction of galactose may contribute to the disease phenotype in susceptible individuals by further depleting uridine diphosphate (UDP)‐galactose, and disrupting glycosylation‐dependent pathways. Build‐up of galactose‐1‐phosphate (Gal‐1‐P) and its metabolites is proposed to contribute to the development of CG complications (Colhoun et al., 2018).

Numerous methods are routinely applied for the detection of mono‐ and disaccharides in human food including spectroscopy, polarimetry, gravimetry, chromatographic, and enzymatic techniques. The performance of the HPAEC/PAD system used in the current study showed acceptable spiking recoveries for galactose and lactose for all matrices in the range 85–112% (Appendix S1 – Table S2). These figures are comparable to recently published data using HPAEC/PAD methods for quantification of fermentable oligo‐, di‐, and monosaccharides in cereals and cereal‐based products (Ispiryan et al., 2019).

When comparing our analysis results with lactose and galactose values in different foods, as cited in McCance and Widdowson (6th Edition) (Paul et al., 1986; Paul & Southgate, 1979) and other analyses (Portnoi & MacDonald, 2015), our results were comparable, particularly in terms of food products such as butter.

Cheese was prioritized for testing, as this patient group is at risk of low bone mineral density and may have suboptimal intake of calcium on a galactose‐restricted diet (van Erven et al., 2017). Furthermore, this analysis aims at validating and consolidating previous analyses of the galactose content of cheeses (Portnoi & MacDonald, 2009; Portnoi & MacDonald, 2016). Our analyses showed that all six dairy‐containing noncheddar hard cheeses that were not labeled “lactose‐free” had a low galactose content (total galactose <25 mg/100 g), while 35% (6 of 17) of mature cheddar cheeses were low in galactose.

The lactose and galactose content of cheese is variable due to differing manufacturing processes, the specific starter bacterial cultures used, and the extent of cheese maturation. Lactose and galactose content in cheese is reduced through two processes: the separation and removal of whey and the fermentation of lactose by bacteria (Portnoi & MacDonald, 2009; Portnoi & MacDonald, 2016). The latter process, often accelerated through the addition of a starter bacteria culture, occurs via the lactase in lactic acid bacteria metabolizing lactose and, in some cases, converting galactose to glucose (Portnoi & MacDonald, 2009; Portnoi & MacDonald, 2016). Fermentation periods and the specific bacterial cultures used affect the lactose content and subsequent galactose content postbacterial metabolism the longer the cheese has matured or ripened. In general, this results in a reduced lactose and galactose content following established methods of hard cheese production (Cogan et al., 1998). Different cheese manufacturers will use different methods, bacterial cultures, and maturation times which all contribute to this variability and which can also change over time if new processing methods are introduced.

For soft and processed cheeses, the curd is separated from the whey and then used immediately (Portnoi & MacDonald, 2009; Portnoi & MacDonald, 2016). Therefore, these cheeses contain a relatively high level of galactose. Milk products (including cheese) labeled “lactose‐free” are in fact lactose‐hydrolyzed milk whereby the lactose is enzymatically converted to glucose and galactose. These cheeses had low lactose content but a much higher galactose content compared to nonhydrolyzed products, as expected. While the processing methods of the cheddar cheeses labeled “lactose‐free” were not available, neither cheese was labeled mature.

The variability in the galactose content of the mature cheddar cheeses analyzed may also reflect differences in processing and packing techniques. More traditional manufacturing processes provide the lowest levels of lactose and galactose, while large‐scale manufactured cheeses are often packed soon after production, with maturation occurring within the package. With the latter method, lactose is not lost within the package and therefore the galactose content of these cheeses tends to be higher (Portnoi & MacDonald, 2016).

Similar analyses in the United Kingdom have expanded the range of cheeses allowed on a low galactose diet (Portnoi & MacDonald, 2009; Portnoi & MacDonald, 2016). These studies also highlight the importance of the manufacturing process to the lactose and galactose content of cheeses, with certain brands of mature cheddar cheeses excluded due a lactose/galactose content >10 mg/100 g. This analysis may further expand the number of suitable cheeses available to people with CG, to include specific brands of mature cheddar cheese, two types of Dutch semihard cow's milk cheese, reduced fat white cheeses, and one vintage cheese from a popular Irish brand.

Similar to cheeses, milk and yogurt products that have been fermented have a lower galactose content(Ohlsson et al., 2017), with longer fermentation times resulting in lower galactose contents (Varga et al., 2006). As manufacturing methods and fermentation periods can vary widely, further analyses are required to ascertain the galactose content of fermented milk products available on the Irish market and their suitability for inclusion in a galactose‐restricted diet.

Whole foods, defined as natural products such as butter and milk, are less likely to experience changes in lactose and extrapolated galactose content over time. In comparison, a more complex food item such as a biscuit or cake containing multiple ingredients and where individual recipes can change without notification to the consumer will be difficult to monitor in terms of estimated lactose and extrapolated galactose content. Furthermore, the higher than expected galactose content of certain products labeled “dairy‐free” indicates the potential impact of cross contamination.

Therefore, it is important to note that it is difficult to know the exact and consistent galactose content of individual processed foods due to the variation in ingredients over time and indeed even within the same batch. However, by reviewing the data available, certain foods could possibly be considered for inclusion in a galactose‐restricted diet in measurable quantities under metabolic clinic supervision with adequate education. These include specific cheeses, packaged pizzas, soups, biscuits, crackers, cakes, pastries, fat spreads, and crisps.

The inclusion of a wider variety of foods for patients with CG could have many benefits, including improved quality of life and nutritional benefits, e.g., inclusion of foods high in calcium and vitamin D. In particular, the consumption of dairy products is recommended to optimize bone health and reduce the risk of osteoporosis (Wallace et al., 2020). Furthermore, this analysis indicates that butter and dairy‐containing spreads, using portion sizes in line with healthy eating guidelines (Flynn et al., 2012; HSE, 2016), may be suitable for CG patients who consume a more liberalized diet. The inclusion of a wider variety of fat spreads in a galactose‐restricted diet would greatly increase food choices, particularly when eating out, and may contribute to patients' dietary variety and quality of life.

The results of our analyses describe the galactose contents of a wide range of foods, thereby contributing to our understanding of the nutritional content of processed foods and providing clinicians with further guidance on the suitability of certain foods for inclusion in a galactose‐restricted diet. Of note, foods chosen for analysis were based on patient preferences and many of these foods were convenience foods high in fat, sugar, and/or salt while some did not contain any lactose‐containing ingredients. As such, this analysis does not reflect a healthy, balanced diet, nor is it a comprehensive investigation of foods potentially high in galactose. Therefore, patient education and advice regarding diet liberalization should aim to ensure a healthy, balanced diet is consumed.

The results of the present study may provide a useful tool to dietitians to aid patients to make more informed choices on food consumption (particularly processed food intake) and dietary pattern. These results could be used to implement advised increases to dietary galactose intake for suitable patients, with close clinical and dietary monitoring.

To allow the safe introduction of slight increases of galactose in the diet, sensitive biomarkers are required (Welling et al., 2017; Treacy et al., 2021), with improved analysis of galactose content in common foods.

Introduction of dietary changes and designing interventions to change dietary behavior is a complex process. It requires extensive education, development of dietary resources, close monitoring of dietary behavior, and availability of financial resources. With the rise of the digital revolution, there has been increased interest in using digital technology for dietary behavioral change as a means of diet‐related management of inborn error of metabolism including CG. It is crucial to advise patients to avoid the risk of exceeding the daily galactose dose that has been medically advised until more detailed biomarkers and outcome studies of moderate galactose liberalization are performed.

## 5 CONCLUSION

This study has enabled the further analysis of food products which could expand the food choice available to patients with CG. This food analysis will be used to develop patient resources which can be individualized to the specific dietary needs of each CG patient. Improved knowledge of the composition of foods and accurate estimation of food ingredients could provide a powerful tool to advise patients on more varied and balanced dietary intake. A limitation of the study is that the analysis of food samples was based on patient preference only. Furthermore, there are some difficulties in terms of variability of results over time related to the dynamics of an evolving food market, e.g., introduction of new brands, discontinuation of other brands, and modification of portion size or content.

## CONFLICT OF INTEREST

All authors declare that they have no conflict of interest.

## Supporting information


Appendix S1
