# Supplementary material for: Determination of the lactose and galactose content of common foods: Relevance to galactosemia
Source: Food Sci Nutr. 2022 Jul 19;10(11):3789–800. doi: 10.1002/fsn3.2976 (PMC9632185; doi:10.1002/fsn3.2976)
Supplement: Supplementary file 1 — Appendix S1 [file FSN3-10-3789-s001.zip › fsn32976-Sup-0001-Tables.docx]

Table 1. Spiking recovery studies.

| **Sample Type** | **Galactose**  **Mean Recovery %** | **Lactose**  **Mean Recovery %** |
| --- | --- | --- |
| Spreads/Butter | 91.59 | 97.21 |
| Lactose Free Cheese | 95.14 | 96.71 |
| Cheddar Cheese | 92.42 | 97.07 |
| Yogurt | 103.96 | 100.13 |
| Salad Cream/ Mayonnaise | 105.07 | 103.65 |
| Soup | 99.45 | 100.1 |
| Biscuits | 107.35 | 99.28 |
| Crackers | 96.54 | 93.87 |
| Cakes | 87.75 | 112.42 |
| Pizza | 93.43 | 94.94 |
| Gravy | 97.15 | 84.78 |
| Potato Crisps | 95.29 | 94.86 |
| Corn Chips | 92.88 | 95 |
| Salami | 111.39 | 92.16 |

Table 2: FAPAS Proficiency Test. Z-scores for Galactose and Lactose

| **Date** | **Round** | **Sample Type** | **Galactose**  **Z-Score** | **Lactose**  **Z-Score** |
| --- | --- | --- | --- | --- |
| Sep-Oct 15 | 25134 | Milkshake Powder | -1.3 | 1.3 |
| Jun-Aug 17 | 25155 | Milkshake Powder | 0.8 | 0.2 |
| Jun-Aug 18 | 25165 | Milkshake Powder | - | 0.7 |
| Jun-Aug 19 | 25175 | Milkshake Powder | 0.5 | 1.4 |
| Jun-Jul 20 | 25185 | Milkshake Powder | 0.0 | 0.7 |

**Table 3.** Average Recovery of Galactose and Lactose in FAPAS Proficiency Tests.

| **Sugar** | **Assigned Value Range**  **g/100g** | **No. of rounds** | **Average Recovery** |
| --- | --- | --- | --- |
| **Galactose** | 0.93-4.23 | 4 | 99.85% |
| **Lactose** | 1.67-16.4 | 5 | 105.21% |

Table 4a. Galactose Food Analysis Results (PAL Galway 2016)- Cheese

| **Lab Ref. No.** | **Sample Description** | **Sample Size**  **(g)** | **Galactose (mg/100g)** | **Lactose (mg/100g)** | **"Released" Galactose (from Lactose) (mg/100g)** | **Total Galactose Value (mg/100g)** | **Portion Weight (g)** | **Total Galactose Value**  **(mg per portion)** |
| --- | --- | --- | --- | --- | --- | --- | --- | --- |
| F148-2016 | Dairy free cheese slices | 200 | <10 | <10 | <5 | <15 | 30 | <5 |
| F149-2016 | Dairy free for pizza cheese | 200 | <10 | <10 | <5 | <15 | 30 | <5 |
| F150-2016 | Dairy free cheddar flavour (Batch 1) | 200 | <10 | <10 | <5 | <15 | 30 | <5 |
| F158-2016 | Irish mature white cheddar (Brand A, Batch 1) | 200 | <10 | <10 | <5 | <15 | 30 | <5 |
| F160-2016 | Reduced fat medium white cheese | 160 | <10 | <10 | <5 | <15 | 30 | <5 |
| F715-2016 | Mature red cheddar (Brand B) | 200 | <10 | <10 | <5 | <15 | 30 | <5 |
| F716-2016 | Dairy free cheddar flavour (Batch 2) | 200 | <10 | <10 | <5 | <15 | 30 | <5 |
| F718-2016 | Mini original natural cheese- 6x 20g | 20 | <10 | <10 | <5 | <15 | 20 | <3 |
| F719-2016 | Rich & mature white Irish cheddar (Brand C) | 200 | <10 | <10 | <5 | <15 | 30 | <5 |
| F720-2016 | Mature white cheddar (Brand D) | 200 | <10 | <10 | <5 | <15 | 30 | <5 |
| F720-2016 | Rich & mature red Irish cheddar (Brand C) | 200 | <10 | <10 | <5 | <15 | 30 | <5 |
| F758-2016 | Irish mature white cheddar (Brand A, Batch 2) | 200 | <10 | <10 | <5 | <15 | 30 | <5 |
| F144-2016 | Original white cheese | 200 | <10 | <10 | <5 | <15 | 30 | <5 |
| F147-2016 | Dutch semihard cheese made from cow's milk (aged 3–12 months) | 160 | <10 | <10 | <5 | <15 | 30 | <5 |
| F146-2016 | Reduced fat mature cheese | 200 | 11 | <10 | <5 | 11 | 30 | 3 |
| F143-2016 | Vintage cheese | 200 | 20 | <10 | <5 | 20 | 30 | 6 |
| F717-2016 | Mature white cheddar (Brand B) | 200 | 32 | <10 | <5 | 32 | 30 | 10 |
| F139-2016 | The fillet of cheddar-fully mature white (Brand E) | 200 | 38 | <10 | <5 | 38 | 30 | 11 |
| F714-2016 | Irish mature white cheddar (Brand A, Batch 3) | 200 | 42 | <10 | <5 | 42 | 30 | 13 |
| F755-2016 | Irish mature white cheddar (Brand A, Batch 4) | 200 | 44 | <10 | <5 | 44 | 30 | 13 |
| F756-2016 | Irish mature white cheddar (Brand A, Batch 5) | 200 | 80 | <10 | <5 | 80 | 30 | 24 |
| F140-2016 | The fillet of cheddar-mature white (Brand E) | 200 | 178 | <10 | <5 | 178 | 30 | 53 |
| F155-2016 | White cheddar cheese | 350 | 193 | <10 | <5 | 193 | 30 | 58 |
| F159-2016 | Irish mature red cheddar (Brand A) | 200 | 216 | <10 | <5 | 216 | 30 | 65 |
| F138-2016 | The fillet of cheddar-mature red (Brand E) | 200 | 219 | <10 | <5 | 219 | 30 | 66 |
| F141-2016 | The fillet of cheddar-fully mature red (Brand E) | 200 | 223 | <10 | <5 | 223 | 30 | 67 |
| F156-2016 | Irish mature red cheddar (Brand A) | 350 | 237 | <10 | <5 | 237 | 30 | 71 |
| F757-2016 | Irish mature white cheddar (Brand A, Batch 6) | 200 | 255 | <10 | <5 | 255 | 30 | 77 |
| F154-2016 | Red cheddar | 350 | 283 | <10 | <5 | 283 | 30 | 85 |
| F152-2016 | Cheese block | 200 | 65 | 1247 | 656 | 721 | 30 | 216 |
| F157-2016 | Red cheddar lactose free (Brand F, Batch 1) | 200 | 824 | <10 | <5 | 824 | 30 | 247 |
| F145-2016 | Red cheddar lactose free (Brand F, Batch 1) | 200 | 908 | <10 | <5 | 908 | 30 | 272 |
| F142-2016 | Single cheese slices | 200 | 50 | 4410 | 2320 | 2370 | 20 | 474 |
| F153-2016 | Cheese slice | 340 | 67 | 4537 | 2386 | 2453 | 17 | 412 |
| F151-2016 | Calcium & vitamins extra mild cheese | 340 | 30 | 4993 | 2626 | 2656 | 30 | 797 |

**Note:** None of these samples are labelled “Lactose free except”: F148-2016 (dairy free), F149-2016 (dairy free), F150-2016 (dairy free) and F716-2016 (dairy free). F145-2016 & F157-2016- Claim <0.1g Lactose /100g

**Table 4b**. **Galactose Food Analysis Results (PAL Galway 2016)-Yogurts**

| **Lab Ref. No.** | **Sample Description** | **Labelled Lactose-free (Yes/No)** | **Sample Size**  **(g)** | **Galactose (mg/100g)** | **Lactose (mg/100g)** | **"Released”Galactose (from Lactose) (mg/100g)"** | **Total Galactose Value (mg/100g)** | **Portion Weight (g)** | **Total Galactose Value**  **(mg per portion)** |
| --- | --- | --- | --- | --- | --- | --- | --- | --- | --- |
| F1495-2016 | Dairy Free Coconut & Lemon- Coconut Milk Yogurt | Yes | 2 x 125 | <10 | 15 | 8 | 8 | 125 | 10 |
| F1496-2016 | Dairy Free Peach and Passionfruit Coconut Milk Yogurt | Yes | 2 x 125 | <10 | <10 | <5 | <15 | 125 | <19 |
| F1497-2016 | Simply Plain Soya yogurt | Yes | 4 x 125 | <10 | <10 | <5 | <15 | 125 | <19 |
| F1499A-2016 | Multipack Soya yogurt (2 x Strawberry-Banana) | Yes | 2 x 125 | <10 | <10 | <5 | <15 | 125 | <19 |
| F1499B-2016 | Multipack Soya yogurt (2 x Peach-Pear) | Yes | 2 x 125 | <10 | <10 | <5 | <15 | 125 | <19 |
| F1498A-2016 | Multipack Soya yogurt (2 x Blueberry) | Yes | 2 x 125 | <10 | 63 | 33 | 33 | 125 | 41 |
| F1498B-2016 | Multipack Soya yogurt (2 x Cherry) | Yes | 2 x 125 | <10 | 94 | 49 | 49 | 125 | 62 |
| F1508-2016 | 0% strained Greek Style Blueberry Yogurt | No | 150 | 511 | 2176 | 1145 | 1656 | 150 | 2483 |
| F1509-2016 | 0% strained Greek Style Strawberry Yogurt | No | 150 | 609 | 2227 | 1171 | 1780 | 150 | 2671 |
| F1502A-2016 | Goats Milk Strawberry Yogurt | No | 2 x 90 | 1795 | 103 | 54 | 1849 | 90 | 1664 |
| F1510-2016 | 0% strained Greek Style Natural Yogurt | No | 150 | 607 | 2477 | 1303 | 1910 | 150 | 2865 |
| F1506-2016 | Strawberry Yogurt | No | 160 | 872 | 1992 | 1048 | 1920 | 160 | 3072 |
| F1501A-2016 | Fromage Frais Raspberry | No | 2 x 85 | 946 | 1879 | 988 | 1934 | 85 | 1644 |
| F1501B-2016 | Fromage Frais Strawberry | No | 2 x 85 | 970 | 1883 | 990 | 1960 | 85 | 1666 |
| F1502B-2016 | Goats Milk Raspberry Yogurt | No | 2 x 90 | 1900 | 131 | 69 | 1969 | 90 | 1772 |
| F1513-2016 | Light Strawberry Yogurt | No | 175 | 849 | 2699 | 1420 | 2269 | 175 | 3970 |
| F1505-2016 | Forest Fruits Yogurt | No | 125 | 757 | 3113 | 1637 | 2394 | 125 | 2993 |
| F1504-2016 | Peach & Strawberry Yogurt | No | 125 | 816 | 3088 | 1624 | 2440 | 125 | 3050 |
| F1503-2016 | Natural Yogurt | No | 125 | 705 | 3832 | 2016 | 2721 | 125 | 3401 |
| F1512-2016 | Low Fat Strawberry Yogurt | No | 500 | 835 | 4348 | 2287 | 3122 | 125 | 3903 |
| F1500-2016 | Fromage Frais Strawberry & Apple Baby | No | 4 x 60 | 727 | 4833 | 2542 | 3269 | 60 | 1961 |
| F1511-2016 | Low Fat Natural Yogurt | No | 500 | 1027 | 4922 | 2589 | 3616 | 125 | 4520 |
| F1507-2016 | Greek Style Natural Yogurt | No | 500 | 1244 | 4541 | 2389 | 3633 | 125 | 4541 |

Table 4c. Galactose Food Analysis Results (PAL Galway 2018)-Cooked Pizza

| **Lab Ref. No.** | **Sample Description** | **Sample Size**  **(g)** | **Galactose (mg/100g)** | **Lactose (mg/100g)** | **"Released" Galactose**  **(from Lactose) (mg/100g)** | **Total Galactose Value (mg/100g)** | **Portion Weight (g)** | **Total Galactose Value**  **(mg per portion)** |
| --- | --- | --- | --- | --- | --- | --- | --- | --- |
| F1251-2018 | Pizza pepperoni-salame (mozzarella cheese (7%) & edam (7%) | 320g | 47 | 7 | 4 | 51 | 160 | 81 |
| F1252-2018 | Pizza Hawaii (mozzarella cheese (7%) & edam (7%) | 355g | 55 | 7 | 4 | 59 | 178 | 104 |
| F1262-2018 | The deep dish 2 four cheese (mozzarella cheese (15%), mature cheddar (4.5%), monterey jack cheese (2%) & emmental (1%) | 310g | 70 | 2 | 1 | 71 | 155 | 110 |
| F1255-2018 | Thin pepperoni pizza (mozzarella cheese with starch (17%) | 340g | 66 | 42 | 22 | 88 | 170 | 150 |
| F1261-2018 | The deep dish 2 pepperoni (mozzarella cheese (15%) | 320g | 103 | 3 | 2 | 105 | 160 | 167 |
| F1250-2018 | Pizza mozzarella (mozzarella cheese (20%) & edam cheese (5%) | 335g | 121 | 2 | 1 | 122 | 168 | 205 |
| F1260-2018 | Take-away stuffed crust loaded pepperoni (mozzarella cheese (12%) | 490g | 84 | 8 | 4 | 88 | 245 | 216 |
| F1249-2018 | Pizza thin base- double pepperoni (mozzarella cheese (14%) | 349g | 69 | 125 | 66 | 135 | 173 | 233 |
| F1248-2018 | Pizza thin base- ham & pineapple (mozzarella cheese (12%) | 387g | 75 | 124 | 65 | 140 | 175 | 245 |
| F1257-2018 | Extra thin stone baked pepperoni & chorizo (mozzarella (14%), cheddar cheese (1.6%) & cheese powder) | 328g | 95 | 119 | 63 | 158 | 164 | 258 |
| F1259-2018 | The thin one-double pepperoni (mozzarella cheese (20%) | 305g | 176 | 2 | 1 | 177 | 153 | 271 |
| F1254-2018 | Thin ham and pineapple pizza (mozzarella cheese (17%) | 365g | 98 | 124 | 65 | 163 | 183 | 299 |
| F1253-2018 | Thin margherita pizza (mozzarella cheese with starch (20%) & cheddar cheese (10%) | 345g | 122 | 121 | 64 | 186 | 173 | 321 |
| F1258-2018 | The thin one- triple cheese (mozzarella cheese (23%), monterey jack cheese (5%) & mature cheddar (3%) | 305g | 214 | 4 | 2 | 216 | 153 | 331 |
| F1247-2018 | Pizza thin base- four cheese (mozzarella cheese (14%), mature cheddar cheese (7%), red cheddar cheese (4.5%) & monterey jack cheese (1%) | 352g | 114 | 159 | 84 | 198 | 170 | 336 |
| F1256-2018 | Deep pan baked deliciously cheesy (mozzarella cheese with starch (21%), cheddar cheese (5%) & monterey jack cheese (3%) | 417g | 93 | 149 | 78 | 171 | 209 | 358 |

Table 4d. Galactose Food Analysis Results (PAL Galway 2018) -Take- Away Pizza

| **Lab Ref. No.** | **Sample Description** | **Sample Size**  **(g)** | **Galactose (mg/100g)** | **Lactose (mg/100g)** | **"Released" Galactose (from Lactose) (mg/100g)** | **Total Galactose Value (mg/100g)** | **Portion Weight***  **(g)** | **Total Galactose Value**  **(mg per portion)** |
| --- | --- | --- | --- | --- | --- | --- | --- | --- |
| F1644-2018 | Regular base ham and pineapple 9" pizza **(franchise 4)** | Loose sample | 130 | 4 | 2 | 132 | 250 | 330 |
| F1643-2018 | Regular base pepperoni 9" pizza **(franchise 4)** | Loose sample | 149 | 4 | 2 | 151 | 250 | 378 |
| F1642-2018 | Regular base) cheese 9" pizza **(Franchise 4)** | Loose sample | 166 | 4 | 2 | 168 | 250 | 420 |
| F1641-2018 | Regular base ham and pineapple 9" pizza **(franchise 3)** | Loose sample | 201 | 12 | 6 | 207 | 250 | 518 |
| F1502-2018 | Ham and pineapple 10" pizza **(franchise 1)** | Loose sample | 90 | 256 | 135 | 225 | 250 | 562 |
| F1640-2018 | (Regular base) pepperoni 9" pizza **(franchise 3)** | Loose sample | 237 | 13 | 7 | 244 | 250 | 610 |
| F1639-2018 | (Regular base) cheese 9"pizza **(franchise 3)** | Loose sample | 268 | 13 | 7 | 275 | 250 | 687 |
| F1501-2018 | Original crust pepperoni 10" pizza (**franchise 1**) | Loose sample | 115 | 323 | 170 | 285 | 250 | 712 |
| F1505-2018 | Classic crust pepperoni 9.5" pizza **(franchise 2)** | Loose sample | 125 | 322 | 169 | 294 | 250 | 736 |
| F1504-2018 | Classic crust ham and pineapple 9.5" pizza **(franchise 2)** | Loose sample | 135 | 328 | 173 | 308 | 250 | 769 |
| F1500-2018 | Original crust cheese and tomato 10" pizza **(franchise 1)** | Loose sample | 125 | 370 | 195 | 320 | 250 | 799 |
| F1503-2018 | Classic crust cheese and tomato 9.5" pizza **(franchise 2)** | Loose sample | 153 | 374 | 197 | 350 | 250 | 874 |

*Portion size taken as 250g for takeaway pizza based on an average pizza size of approximately 500g

Table 4e. Galactose Food Analysis Results (PAL Galway 2017-2018)- Soup

| **Lab Ref. No.** | **Sample Description** | **Sample Size**  **(g)** | **Galactose (mg/100g)** | **Lactose (mg/100g)** | **"Released" Galactose (from Lactose) (mg/100g)** | **Total Galactose Value (mg/100g)** | **Portion Weight (g)** | **Total Galactose Value**  **(mg per portion)** |
| --- | --- | --- | --- | --- | --- | --- | --- | --- |
| F30-2018 | Chicken broth *(No Milk Ingredients)* | 400 | 3 | <2 | <1 | 3 | 200 | 6 |
| F31-2018 | Chicken & vegetable *(no milk ingredients)* | 400 | 3 | <2 | <1 | 3 | 200 | 6 |
| F36-2018 | classic vegetable *(no milk ingredients)* | 400 | 3 | <2 | <1 | 3 | 200 | 6 |
| F1419-2017 | Wholesome Chicken & Vegetable Soup *(Cream & Butter)* | 400 | <60 | 70 | 37 | 37 | 200 | 74 |
| F1421-2017 | Slow cooked chicken & vegetable soup *(Fresh Irish Cream & Irish Creamery Butter)* | 400 | 2 | 90 | 47 | 49 | 200 | 99 |
| F1418-2017 | Mushroom Soup *(Cream & Butter)* | 400 | <30 | 150 | 79 | 79 | 200 | 158 |
| F1420-2017 | Smooth vegetable with creme fraiche & garden herbs soup *(Cream, Butter & Crème Fraiche)* | 400 | <30 | 160 | 84 | 84 | 200 | 168 |
| F1417-2017 | Creamy Tomato & Basil Soup *(Cream & Butter)* | 400 | <60 | 230 | 121 | 121 | 200 | 242 |
| F35-2018 | Cream of tomato & basil *(dried skimmed milk, milk proteins & cream)* | 400 | 6 | 440 | 231 | 237 | 200 | 475 |
| F34-2018 | cream of tomato *(milk, butter, double cream & skimmed milk powder)* | 400 | 3 | 527 | 277 | 280 | 200 | 560 |
| F33-2018 | Cream of tomato *(milk, butter, double cream & skimmed milk powder)* | 400 | 2 | 532 | 280 | 282 | 200 | 564 |
| F1424-2017 | Potato, leek & bacon soup *(Cream, Flavourings (contain Lactose), Buttermilk powder & Skimmed Milk Powder)* | 390 | 82 | 543 | 286 | 368 | 200 | 735 |
| F32-2018 | Cream of chicken *(double cream & skimmed milk powder)* | 400 | 14 | 751 | 395 | 409 | 200 | 818 |
| F29-2018 | Cream of chicken soup *(Cream, Dried Skimmed Milk, Milk proteins)* | 400 | 20 | 1043 | 549 | 569 | 200 | 1137 |
| F1423-2017 | Cream of mushroom soup *(Dried Skimmed Milk, Cream & Milk proteins)* | 400 | 40 | 1550 | 815 | 855 | 200 | 1711 |

Table 4f. Galactose Food Analysis Results (PAL Galway 2017)- Biscuits

| Lab Ref. No. | **Sample Description** | **Sample Size**  **(g)** | **Galactose (mg/100g)** | **Lactose (mg/100g)** | **"Released" Galactose (from Lactose) (mg/100g)** | **Total Galactose Value (mg/100g)** | **Biscuit Weight (g)** | **Total Galactose Value**  **(mg per Biscuit)** |
| --- | --- | --- | --- | --- | --- | --- | --- | --- |
| F1543-2017 | Rich Tea Classic *(No Milk Declared)* | 300 | <11 | 11 | 6 | 6 | 8.3 | <0.5 |
| F1534-2017 | Bourbon Creams *(May contain traces of Milk)* | 150 | <11 | <11 | <6 | <17 | 12.5 | <2.1 |
| F1537-2017 | Digestives *(Dried Skimmed Milk)* | 250 | <11 | 32 | 17 | 17 | 14.7 | 2.5 |
| F1538-2017 | Pink Wafers *(May contain traces of Milk)* | 100 | <11 | <11 | <6 | <17 | 7.14 | <1.2 |
| F1540-2017 | Oat Biscuit  *(May contain Milk)* | 300 | <11 | <11 | <6 | <17 | 15.2 | <2.6 |
| F1546-2017 | Ginger Nut *(May contain traces of Milk)* | 250 | <11 | <11 | <6 | <17 | 9.8 | <1.7 |
| F1545-2017 | Fig rolls *('Produced in a factory handling milk but on a different line')* | 200 | <11 | 41 | 22 | 22 | 16.7 | 3.7 |
| F1535-2017 | Orange & chocolate Cakes *(Plain Chocolate contains Butteroil (Milk))* | 244 | <11 | 159 | 84 | 84 | 12.2 | 10.3 |
| F1536-2017 | Custard Creams *(Whey Powder Milk)* | 150 | <11 | 177 | 93 | 93 | 12.5 | 11.6 |
| F1541-2017 | Ginger flavoured biscuits with a mallow centre *(May contain traces of Milk)* | 300 | 45 | 126 | 66 | 111 | 15 | 16.7 |
| F1542-2017 | Shortcake biscuits with jam centre *(Whey or Whey derivatives (Milk))* | 140 | 14 | 1103 | 580 | 594 | 17.5 | 104 |
| F1544-2017 | Milk chocolate digestives *(Milk Chocolate, Dried Skimmed Milk, Dried Whey (Milk), Butteroil (Milk)* | 200 | <11 | 1929 | 1015 | 1015 | 16.7 | 169.5 |
| F1539-2017 | Chocolate wafer fingers with coffee flavour | 186.3 | 108 | 5839 | 3071 | 3179 | 20.7 | 658.1 |
| F663-2018 | 6 Viennese Whirls *(Whey Powder (Milk) & Milk Proteins)* | 6 x28 | <2 | 30 | 16 | 16 | 28 | 4 |

Table 4g. Galactose Food Analysis Results (PAL Galway 2018)- Crackers

| Lab Ref. No. | **Sample Description** | **Sample Size**  **(g)** | **Galactose (mg/100g)** | **Lactose (mg/100g)** | **"Released" Galactose (from Lactose) (mg/100g)** | **Total Galactose Value (mg/100g)** | **Cracker Weight (g)** | **Total Galactose Value (mg per Cracker)** |
| --- | --- | --- | --- | --- | --- | --- | --- | --- |
| F186-2018 | Oatcake *(Manufactured on equipment that handles Milk*) | 291 | <2 | <2 | <1 | <3 | 10.39 | <0.3 |
| F183-2018 | Salted Savoury Snack Biscuits *(May contain traces of Milk)* | 100 | 2 | 6 | 3 | 5 | 3.6 | 0.18 |
| F182-2018 | Cream Crackers *(No Milk Declared)* | 200 | 7 | 5 | 3 | 10 | 8 | 0.8 |
| F187-2018 | Crackerbread *(May contain Milk)* | 200 | <2 | 22 | 12 | 12 | 5 | 0.6 |
| F185-2018 | Table Water Biscuits *(No Milk Declared*) | 125 | 26 | 16 | 8 | 34 | 3.4 | 1.16 |
| F184-2018 | Cheese Flavoured Savoury Crackers *(Cheese powder 5.4%)* | 200 | 9 | 210 | 110 | 119 | 3.57 | 4.25 |

Table 4h. Galactose Food Analysis Results (PAL Galway 2020)-Cake and Pastries.

| **Lab Ref. No.** | **Sample Description** | **Sample Size**  **(g)** | **Galactose (mg/100g)** | **Lactose (mg/100g)** | **"Released" Galactose (from Lactose) (mg/100g)** | **Total Galactose Value (mg/100g)** | **Portion Weight (g)** | **Total Galactose Value**  **(mg per portion)** |
| --- | --- | --- | --- | --- | --- | --- | --- | --- |
| F661-2018 | Bramley Apple Pies *(Milk Proteins & Whey Powder)* | 6 x 59 | <2 | 20 | 11 | 11 | 59 | 6 |
| F670-2018 | Pain au Chocolate (2) *(Loose Sample)* | 137.49 | 4 | 113 | 59 | 63 | 69 | 44 |
| F660-2018 | Queen Cakes (12) *(Whey Protein (milk), Dried Skimmed Milk, Whey Protein Concentrate (milk) & Buttermilk Powder)* | 330 | 13 | 413 | 217 | 230 | 27 | 62 |
| F654-2018 | Croissants (2) *(Loose sample)* | ~119.34 | 9 | 193 | 102 | 111 | 60 | 66 |
| F662-2018 | 5 Mini Battenberg *(Skimmed Milk Powder)* | 5 x 32 | <2 | 422 | 222 | 222 | 32 | 71 |
| F657-2018 | Ripple Swiss Roll *(Skimmed Milk Powder)* | 205 | 4 | 415 | 218 | 222 | 37 | 82 |
| F655-2018 | 8 All Butter Croissants *(Concentrated Butter (18%), Skimmed Milk Powder & Milk Proteins)* | 320 | 11 | 432 | 227 | 238 | 40 | 95 |
| F659-2018 | Ginger Sticky Pudding Cake *(Dried Whey(milk) & Milk Protein)* | 232.2 | 35 | 721 | 379 | 414 | 25.8 | 107 |
| F671-2018 | Madeira cake *(Whey Powder(milk), Buttermilk & Skimmed Milk Powder)* | 310 | 16 | 583 | 307 | 323 | 39 | 126 |
| F669-2018 | Croissants (2) *(Loose Sample)* | ~121.85 | 12 | 435 | 229 | 241 | 61 | 147 |
| F656-2018 | Pineapple Swiss Roll *(Skimmed Milk Powder)* | 195 | 10 | 868 | 457 | 467 | 36 | 168 |
| F658-2018 | Raspberry Sponge *(Milk proteins)* | 228 | 11 | 895 | 471 | 482 | 38 | 183 |
| F673-2018 | 12 Iced Queen Cakes *(Skimmed Milk Powder, Whole Milk Powder, Lactose(milk) & Whey Powder(milk)* | 335 | <2 | 1600 | 842 | 842 | 29 | 244 |
| F672-2018 | Blueberry Muffin (1) *(Loose Sample)* | 102.77 | 51 | 521 | 274 | 325 | 103 | 335 |

Table 4i. Galactose Food Analysis Results (PAL Galway 2017)- Mayonnaise and Salad Creams

| **Lab Ref. No.** | **Sample Description** | **Sample Size**  **(g)** | **Galactose (mg/100g)** | **Lactose (mg/100g)** | **"Released" Galactose (from Lactose) (mg/100g)** | **Total Galactose Value (mg/100g)** | **Portion Weight (g)** | **Total Galactose Value**  **(mg per portion)** |
| --- | --- | --- | --- | --- | --- | --- | --- | --- |
| F957-2017 | Real mayonnaise *(no milk ingredients)* | 470 | <25 | <25 | <13 | <38 | 30 | <11 |
| F961-2017 | Light mayonnaise *(no milk ingredients)* | 504 | <25 | <25 | <13 | <38 | 30 | <11 |
| F962-2017 | Real mayonnaise *(no milk ingredients)* | 475 | <25 | <25 | <13 | <38 | 30 | <11 |
| F964-2017 | Real mayonnaise *(no milk ingredients)* | 200 | <25 | <25 | <13 | <38 | 30 | <11 |
| F958-2017 | Light mayonnaise *(cream powder)* | 432 | <100 | 155 | 82 | 82 | 30 | 24 |
| F956-2017 | Light mayonnaise *(milk cream powder)* | 500 | <100 | 159 | 84 | 84 | 30 | 25 |
| F27-2018 | Salad cream (no milk ingredients) | 315 | <25 | <25 | <13 | <38 | 30 | <11 |
| F28-2018 | Salad cream (no milk ingredients) | 510 | <25 | <25 | <13 | <38 | 30 | <11 |

Table 4j. Galactose Food Analysis Results (PAL Galway 2016) -Fat Spreads & Butter

| **Lab Ref. No.** | **Sample Description** | **Sample Size (g)** | **Galactose (mg/100g)** | **Lactose (mg/100g)** | **“Released”Galactose**  **(from Lactose) (mg/100g)** | **Total Galactose Value (mg/100g)** | **Portion Weight (g)** | **Total Galactose (mg per portion)** |
| --- | --- | --- | --- | --- | --- | --- | --- | --- |
| F821-2016 | For biscuits & Pastry" (No dairy listed in ingredients list) | 250 | <10 | <10 | <5 | <15 | 10 | <1.5 |
| F814-2016 | Light (Buttermilk listed as an ingredient) | 250 | <10 | 23 | 12.1 | 12 | 10 | 1.2 |
| F819-2016 | Original (Reconstituted Buttermilk (3%) listed as an ingredient) | 250 | <10 | 124 | 65.2 | 65 | 10 | 6.5 |
| F813-2016 | Original (Buttermilk listed as an ingredient) | 250 | <10 | 217 | 114 | 114 | 10 | 11.4 |
| F818-2016 | Fat spread (Buttermilk listed as an ingredient) | 500 | <10 | 228 | 120 | 120 | 10 | 12 |
| F815-2016 | Buttery taste (Buttermilk (6 %) listed as an ingredient) | 500 | <10 | 255 | 134 | 134 | 10 | 13.4 |
| F816-2016 | Light (whey powder listed as an ingredient) | 250 | <10 | 320 | 168 | 168 | 10 | 16.8 |
| F817-2016 | Fat spread (Buttermilk (10%) listed as an ingredient) | 500 | <10 | 438 | 230 | 230 | 10 | 23 |
| F820-2016 | Butter (Cream listed as an ingredient) | 227 | <10 | 634 | 333 | 333 | 10 | 33.3 |

Table 4k. Galactose Food Analysis Results (PAL Galway 2021)- potato crisps & tortilla/chips.

| **Lab Ref. No.** | **Category** | **Sample Description** | **Sample Size**  **(g)** | **Galactose (mg/100g)** | **Lactose (mg/100g)** | **"Released" Galactose (from Lactose) (mg/100g)** | **Total Galactose Value (mg/100g)** | **Typical Portion Size**  **(g)** | **Total Galactose Value (mg per portion)** |
| --- | --- | --- | --- | --- | --- | --- | --- | --- | --- |
| F508-2021 | Potato Crisps | Cheese & Onion (Vegetarian Cheese Powder(milk) & Flavouring (Contains milk)) | 12 x 25g | <5 | 9 | 5 | 5 | 25 | 1 |
| F886-2020 | Corn Chips | Chilli Heatwave (No Milk Ingredients) | 150 | <5 | <5 | <3 | <8 | 30 | <2 |
| F890-2020 | Potato Crisps | Cheese & Onion (No Milk Ingredients) | 6 x 25g | <5 | <5 | <3 | <8 | 25 | <2 |
| F507-2021 | Potato Crisps | Cheese & Onion (No Milk Ingredients) | 6 x 25g | <5 | <5 | <3 | <8 | 25 | <2 |
| F883-2020 | Potato Crisps | Cheddar Cheese & Spring Onion (No Milk Ingredients) | 135 | <5 | <5 | <3 | <8 | 34 | <3 |
| F885-2020 | Potato Crisps | Salt & Malt Vinegar (No Milk Ingredients) | 135 | <5 | <5 | <3 | <8 | 34 | <3 |
| F889-2020 | Potato Crisps | Cheese & Onion (Cheese powder (milk)) | 6 x 25g | <5 | 15 | 8 | 8 | 25 | 2 |
| F891-2020 | Savoury Snacks | Cheese & Onion (Lactose(milk) & Cheese powder (milk)) | 200 | <5 | 24 | 13 | 13 | 30 | 4 |
| F892-2020 | Savoury Snacks | Sour Cream & Onion (Sour Cream Powder(milk), Flavourings (milk), Sweet Whey Powder (milk) & Milk proteins) | 200 | <5 | 89 | 47 | 47 | 30 | 14 |
| F510-2021 | Potato Crisps | Cheese & Onion (Whey Powder(milk), Flavouring (contains milk), Mature Cheddar Cheese powder (milk) & Cheese powder(milk)) | 6 x 25g | <5 | 280 | 147 | 147 | 25 | 37 |
| F887-2020 | Corn Chips | Cool Original (Milk protein, Flavourings (contains milk) & Cheese Powder (from milk)) | 150 | <5 | 346 | 182 | 182 | 30 | 55 |
| F516-2021 | Potato Snacks | Sour Cream & Onion Baked Snacks (Soured Cream Powder (milk)& Whey Powder (milk)) | 150 | <14 | 483 | 254 | 254 | 30 | 76 |
| F888-2020 | Corn Chips | Tangy Cheese (Buttermilk Powder, Skimmed Milk Powder, Flavourings (from milk), Whey Powder (from milk), Cheese powder (from milk) & Milk Protein) | 150 | 17 | 970 | 510 | 527 | 30 | 158 |
| F515-2021 | Potato Crisps | Cheese & Onion (Dried Whey (milk), Lactose(milk) & Natural Flavouring (milk)) | 6 x 25g | 8 | 1001 | 527 | 535 | 25 | 134 |
| F511-2021 | Tortilla Chips | Cool Flavour (Whey Powder(milk), Buttermilk Powder(milk), Flavouring (contains milk), Dried Skimmed Milk, Cheddar Cheese Powder(milk) & Cheese powder(milk)) | 200 | 6 | 1089 | 573 | 579 | 25 | 145 |
| F512-2021 | Potato Crisps | Cheese & Onion (Dried Milk whey, Lactose (from milk), Flavouring (contains milk) & Cheese powder (from milk)) | 175 | <5 | 1162 | 611 | 611 | 30 | 183 |
| F884-2020 | Potato Crisps | Sour Cream & Onion (Skimmed Milk Powder, Whey Powder(milk), Yoghurt Powder(milk), Buttermilk Powder, Cream Powder(milk) & Flavourings(milk)) | 135 | 5 | 1338 | 704 | 709 | 34 | 241 |
| F513-2021 | Potato Snacks | Oven baked Cheese & Onion (Whey permeate (from milk), Flavouring (contains milk), Whey protein (from milk), Dried cheese (0.1%) (from milk) & Skimmed Milk powder) | 6 x 25g | <5 | 1422 | 748 | 748 | 25 | 187 |
| *F607-2021 | Tortilla Chips | Cool Flavour (Whey Powder(milk), Buttermilk Powder, Yogurt powder (milk), Cream Powder(milk), Flavourings (contains milk) & Cheese powder(milk)) | 200 | 18 | 1562 | 822 | 840 | 40 | 336 |
| *F509-2021 | Tortilla Chips | Cool Flavour (Whey Powder(milk), Buttermilk Powder, Yogurt powder (milk), Cream Powder(milk), Flavourings (contains milk) & Cheese powder(milk)) | 200 | 21 | 1797 | 945 | 966 | 40 | 386 |
| F514-2021 | Potato Crisps | Salt & Vinegar (Lactose(milk) & Dried Skimmed Milk) | 6 x 25g | <5 | 2610 | 1373 | 1373 | 25 | 343 |

***Different Batch/Lot No. of the same product.**

Table 4l. Galactose Food Analysis Results (PAL Galway 2021)-Salami.

| **Lab Ref. No.** | **Category** | **Sample Description** | **Sample Weight (g)** | **Galactose (mg/100g)** | **Lactose (mg/100g)** | **"Released" Galactose (from Lactose) (mg/100g)** | **Total Galactose Value (mg/100g)** | **Typical Portion Size(g)** | **Total Galactose Value (mg per portion)** | |
| --- | --- | --- | --- | --- | --- | --- | --- | --- | --- | --- |
| F695-2021 | Salami | Deli- Italian Milano Salami (16 Slices) (No Milk Ingredients) | 90 | <5 | <5 | <3 | <8 | 18 | < | 1 |
| F712-2021 | Salami | Smoked Salami Slices (No Milk Ingredients) | 150 | <5 | <5 | <3 | <8 | 15 | < | 1 |
| F713-2021 | Salami | German Style Peppered Salami Slices (No Milk Ingredients) | 110 | <5 | <5 | <3 | <8 | 30 | < | 2 |
| F714-2021 | Salami | Stores-German Style Salami (No Milk Ingredients) | 100 | <5 | <5 | <3 | <8 | 30 | < | 2 |

Table 4m. Galactose Food Analysis Results (PAL Galway 2020)- Gravy

| **Lab Ref. No.** | **Category** | **Sample Description** | **Sample Size**  **(g)** | **Galactose (mg/100g)** | **Lactose (mg/100g)** | **"Released" Galactose (from Lactose) (mg/100g)** | **Total Galactose Value (mg/100g)** | **Manu-facturers Prep Instructions** | **Portion Volume (ml)** | **Total Galactose Value**  **(mg per portion)** |
| --- | --- | --- | --- | --- | --- | --- | --- | --- | --- | --- |
| F138-2020 | Gravy granules | Meat gravy granules (no milk ingredients) | 300 | <5 | <5 | <3 | <8 | ~20g + 280ml  (1/15 diln) | 50 | <0.3 |
| F139-2020 | Gravy granules | Chicken gravy granules (no milk ingredients) | 300 | <5 | <5 | <3 | <8 | ~20g + 280ml  (1/15 diln) | 50 | <0.3 |
| F140-2020 | Gravy granules | Onion gravy granules (no milk ingredients) | 300 | <5 | <5 | <3 | <8 | ~20g + 280ml  (1/15 diln) | 50 | <0.3 |
| F141-2020 | Gravy granules | Gravy granules for meat dishes (no milk ingredients) | 300 | <5 | <5 | <3 | <8 | ~20g + 280ml  (1/15 diln) | 50 | <0.3 |
| F142-2020 | Gravy granules | Gravy granules for chicken dishes (flavourings (contain milk)) | 300 | <5 | <5 | <3 | <8 | ~20g + 280ml  (1/15 diln) | 50 | <0.3 |
| F143-2020 | Gravy granules | Onion gravy granules (no milk ingredients) | 300 | <5 | <5 | <3 | <8 | ~20g + 280ml  (1/15 diln) | 50 | <0.3 |
| F144-2020 | Gravy granules | Gravy granules- 25% less salt (no milk ingredients) | 170 | <5 | <5 | <3 | <8 | ~20g + 280ml  (1/15 diln) | 50 | <0.3 |
| F145-2020 | Gravy granules | Vegetable gravy granules- (no milk ingredients) | 170 | <5 | <5 | <3 | <8 | ~20g + 280ml  (1/15 diln) | 50 | <0.3 |
| F146-2020 | Gravy granules | Chicken gravy granules- (flavourings (contain milk) | 170 | <5 | <5 | <3 | <8 | ~20g + 280ml  (1/15 diln) | 50 | <0.3 |
| F147-2020 | Gravy granules | Turkey gravy granules- (flavourings (contain milk) | 170 | <5 | <5 | <3 | <8 | ~20g + 280ml  (1/15 diln) | 50 | <0.3 |
| F148-2020 | Gravy granules | Gravy granules- (no milk ingredients) | 170 | <5 | <5 | <3 | <8 | ~20g + 280ml  (1/15 diln) | 50 | <0.3 |
| F149-2020 | Gravy granules | Onion gravy granules- (no milk ingredients) | 170 | <5 | <5 | <3 | <8 | ~20g + 280ml  (1/15 diln) | 50 | <0.3 |
| F150-2020 | Gravy | Turkey gravy- (no milk ingredients) | 200 | <5 | <5 | <3 | <8 | ~20g + 280ml  (1/15 diln) | 50 | <0.3 |
| F151-2020 | Gravy | Beef gravy- (no milk ingredients) | 200g | <5 | <5 | <3 | <8 | ~20g + 280ml  (1/15 diln) | 50 | <0.3 |
| F155-2020 | Gravy granules | Gravy granules- (no milk ingredients) | 170g | <5 | <5 | <3 | <8 | ~20g + 280ml  (1/15 diln) | 50 | <0.3 |
| F152-2020 | Gravy granules | Beef gravy granules- (no milk ingredients) | 200g | <5 | <5 | <3 | <8 | ~20g + 280ml  (1/15 diln) | 75 | <0.4 |
| F153-2020 | Gravy granules | Chicken gravy granules- (no milk ingredients) | 200g | <5 | <5 | <3 | <8 | ~20g + 280ml  (1/15 diln) | 75 | <0.4 |
| F156-2020 | Gravy | Gravy rich original- (milk proteins) | 375g | <5 | <5 | <3 | <8 | ~28g + 280ml  (1/11 diln) | 50 | <0.4 |
| F154-2020 | Gravy | Gravy gluten free- (lactose & milk proteins) | 185g | 28 | 495 | 260 | 288 | ~25g + 300ml  (1/13 diln) | 100 | **22.2** |
