# Supplementary material for: Determination of the lactose and galactose content of common foods: Relevance to galactosemia
Source: Food Sci Nutr. 2022 Jul 19;10(11):3789–800. doi: 10.1002/fsn3.2976 (PMC9632185; doi:10.1002/fsn3.2976)
Supplement: Supplementary file 1 — Appendix S1 [file FSN3-10-3789-s001.zip › fsn3_2976-sup-0001-supinfo.doc]

**Galactosaemia phone survey questions**

1. Are you taking foods containing lactose and/or galactose?

2. What foods containing galactose are you eating?

3. And how often?

4. What foods do you still avoid?

5. Which of the following foods would you like to eat if found suitable?

LIST FOOD Types in table with YES/NO column

| **Food Type** | **YES/NO** |
| --- | --- |
| **Fat Spread** |  |
| **Cheese** |  |
| **Biscuits** |  |
| **Soups** |  |
| **Sauces** |  |
| **Cakes** |  |
| **Crisps** |  |
| **Sausages** |  |
| **Condiments** |  |
| **Pizza** |  |
| **Burgers/Meats** |  |
| **Fast Food** |  |
| **Ready Meals** |  |
| **Alcohol** |  |
| **Other** |  |
| **Milk** |  |
| **Chocolate** |  |

6. What brand names of foods on this list do you have a preference to be analysed?

| **Food Type** | **Brand Name Suggestions** |
| --- | --- |
| **Fat Spread** |  |
| **Cheese** |  |
| **Biscuits** |  |
| **Soups** |  |
| **Sauces** |  |
| **Cakes** |  |
| **Crisps** |  |
| **Sausages** |  |
| **Condiments** |  |
| **Pizza** |  |
| **Burgers/Meats** |  |
| **Fast Food** |  |
| **Ready Meals** |  |
| **Alcohol** |  |
| **Other** |  |
| **Milk** |  |
| **Chocolate** |  |

7. Would you be interested in joining a focus group looking at future foods to be analysed?

8. It YES Would you be available to attend meetings in Dublin or could you join a meeting by phone if a facility can be arranged?

9. Do you want feedback on the results of this phone survey?

10. Request email address and consent to use it for future contact in relation to food analysis

Thank You
